# H_2_O_2_ Signature and Innate Antioxidative Profile Make the Difference Between Sensitivity and Tolerance to Salt in Rice Cells

**DOI:** 10.3389/fpls.2018.01549

**Published:** 2018-10-23

**Authors:** Elide Formentin, Cristina Sudiro, Maria Beatrice Ronci, Vittoria Locato, Elisabetta Barizza, Piergiorgio Stevanato, Bushra Ijaz, Michela Zottini, Laura De Gara, Fiorella Lo Schiavo

**Affiliations:** ^1^Department of Biology, University of Padova, Padova, Italy; ^2^Unit of Food Science and Human Nutrition, Campus Bio-Medico University of Rome, Rome, Italy; ^3^Department of Agronomy, Food, Natural Resources, Animal and Environment, DAFNAE, University of Padova, Padova, Italy; ^4^Department of Biosciences, COMSATS University Islamabad, Islamabad, Pakistan

**Keywords:** K^+^ transporters, *Oryza sativa*, reactive oxygen species, redox homeostasis, salt stress, signaling

## Abstract

Salt tolerance is a complex trait that varies between and within species. H_2_O_2_ profiles as well as antioxidative systems have been investigated in the cultured cells of rice obtained from Italian rice varieties with different salt tolerance. Salt stress highlighted differences in extracellular and intracellular H_2_O_2_ profiles in the two cell cultures. The tolerant variety had innate reactive oxygen species (ROS) scavenging systems that enabled ROS, in particular H_2_O_2_, to act as a signal molecule rather than a damaging one. Different intracellular H_2_O_2_ profiles were also observed: in tolerant cells, an early and narrow peak was detected at 5 min; while in sensitive cells, a large peak was associated with cell death. Likewise, the transcription factor salt-responsive ethylene responsive factor 1 (TF SERF1), which is known for being regulated by H_2_O_2_, showed a different expression profile in the two cell lines. Notably, similar H_2_O_2_ profiles and cell fates were also obtained when exogenous H_2_O_2_ was produced by glucose/glucose oxidase (GOX) treatment. Under salt stress, the tolerant variety also exhibited rapid upregulation of K^+^ transporter genes in order to deal with K^+^/Na^+^ impairment. This upregulation was not detected in the presence of oxidative stress alone. The importance of the innate antioxidative profile was confirmed by the protective effect of experimentally increased glutathione in salt-treated sensitive cells. Overall, these results underline the importance of specific H_2_O_2_ signatures and innate antioxidative systems in modulating ionic and redox homeostasis for salt stress tolerance.

## Introduction

Soil salinization is a major constraint on crop production worldwide and affects at least 33% of arable lands. In addition, more areas are expected to deteriorate in the coming years due to global climate changes (Zhu, 2001; Tester and Davenport, 2003; FAO^1^). Plants differ greatly in their tolerance to salinity. This stress has two different components, osmotic and ionic, both affecting plant cell metabolism (Gupta and Huang, 2014). Unfortunately, most crop species cannot withstand high concentrations of salt, and rice (*Oryza sativa* L.) is the most salt-sensitive cereal species (Flowers and Yeo, 1995).

Soil salinity imposes two primary stresses on plants: firstly osmotic stress, and later ionic stress arises when Na^+^ concentrations reach toxic levels inside the cells (Munns and Tester, 2008). To deal with this adverse condition, plants have evolved a range of physiological and metabolic responses, activating many stress-responsive genes and synthesizing diverse functional proteins and metabolites through a complex signal transduction network (Hirayama and Shinozaki, 2010).

Long-term responses, such as the production of compatible solutes or the regulation of ion channels/transporters involved in the maintenance of a high cytosolic (cyt) [K^+^]/[Na^+^], have been reported as important features for acquiring salt tolerance (Deinlein et al., 2014).

The molecular processes controlling early salt stress perception and signaling are not yet fully understood. High salinity is known to induce the formation of reactive oxygen species (ROS) within plant cells (Gill and Tuteja, 2010; Miller et al., 2010; Gupta and Huang, 2014) at very early response stages (e.g., a few minutes in rice roots, Hong et al., 2009; Formentin et al., 2018).

While ROS can cause oxidative stress, several studies have shown that ROS also play a key role in plants as signal molecules (Foyer and Noctor, 2016; Sewelam et al., 2016; Mittler, 2017). ROS-mediated signaling is controlled through a delicate balance between its production and scavenging. The biological outcome of ROS signaling is heavily related to the chemical identity of ROS, the intensity and subcellular localization of the signal, and is dose dependent (Gechev et al., 2002; de Pinto et al., 2006). Salt-induced ROS are predominantly represented by H_2_O_2_ (Pang and Wang, 2008). Low doses of H_2_O_2_ have been shown to induce protective mechanisms and acclimation responses against oxidative and various abiotic stresses (Gechev et al., 2002; Gill and Tuteja, 2010; Pucciariello et al., 2012; Locato et al., 2018). Elevated concentrations of ROS, alone or in combination with other molecules, induced by several stresses can trigger programmed cell death (PCD; de Pinto et al., 2006; De Michele et al., 2009; Locato et al., 2016; Locato and De Gara, 2018).

On the other hand, to prevent oxidative damage induced by the high production of ROS, plants have evolved enzymatic and non-enzymatic antioxidative systems, which are crucial for ROS homeostasis by controlling the levels of ROS inside the cells (Gill and Tuteja, 2010).

In *Arabidopsis* and rice exposed to salt stress, ROS release depends on the activity of NADPH oxidases (NOXs) of the respiratory burst oxidase homolog protein C-like (RBOH) family (Hong et al., 2009; Ma et al., 2012). Thus, H_2_O_2_ production may initiate an early signal cascade that triggers salt response mechanisms. A signal transduction cascade has been proposed in which a mitogen-activated protein kinase (MAPK) cascade and downstream TFs represent key regulatory components of ROS signaling (Pang and Wang, 2008; Sewelam et al., 2016). Schmidt et al. (2013) identified a SERF1 in rice as a TF that regulates ROS-dependent signaling during the initial response to salt stress.

To the best of our knowledge, few studies have focused on intraspecific salt tolerance mechanisms comprising both the regulation of cell redox homeostasis and ionic balance under salinity (Chen et al., 2013; Cao et al., 2015). An increase in the understanding of new salinity tolerance mechanisms, particularly in crops, is required in order to combine all tolerance mechanisms in a new variety with a high level of salt tolerance (Yeo et al., 1990). Indeed, although *Oryza sativa* is a salt-sensitive species, few salt tolerance traits have been identified in tolerant varieties (Gregorio et al., 2002; Ismail et al., 2007; Mohammadi-Nejad et al., 2010).

The study reported in this paper was performed on suspension cell cultures obtained from the seeds of two Italian rice varieties showing contrasting salt sensitivity, Baldo (B) and Vialone Nano (VN). Suspension cell cultures have been widely used to investigate the physiological and molecular mechanisms involved in plant responses to abiotic stress (Vera-Estrella et al., 1999; Locato et al., 2008; Hamaji et al., 2009; Liu et al., 2013; Sgobba et al., 2015; Wang et al., 2016). Plant suspension cell cultures offer a simplified model for the study of cellular and molecular processes. Their main advantage is that a relatively homogenous population provides a rapid and uniform response to external stimuli, avoiding the complications that arise from using the whole plant. Therefore, the timeline of cellular events following a particular stimulus can be addressed with a very high accuracy. Several parameters of cell growth, senescence and death have also been precisely defined in suspension cell cultures (Carimi et al., 2004; de Pinto et al., 2002, 2006; Zottini et al., 2006).

We investigated the role of H_2_O_2_ as a component of the signaling pathway induced by salt stress in the two rice cell cultures showing contrasting salt sensitivity.

Our results show that the fine-tuning of H_2_O_2_ scavenging, through efficient antioxidative systems, is critical in shaping an effective H_2_O_2_ profile to induce an adaptive response in salt tolerant cells.

## Materials and Methods

### Plant Material and Salt Treatment

Experiments were conducted with non-embryogenic suspension cell cultures of two rice (*Oryza sativa* spp. *japonica*, L.) varieties, Baldo and Vialone Nano. Briefly, mature seeds were dehusked and sterilized in 3.5% bleach with the addition of a drop of TWEEN20 for 20 min in a giratory shaker, followed by 5 min with 3.5% bleach. After five washes in distilled water, seeds were sawn in solid N6 medium [30 g L^-1^ sucrose, Chu (N6) Medium Salt Mixture and vitamins (Duchefa) and 2 mg L^-1^ 2,4-D, pH 5.8] in dark conditions and moved to new plates every 2 weeks. After few months, the callus was friable, and ready to be transferred in liquid medium. The resulting cells were grown at 25°C under dark conditions on a gyratory shaker in liquid N6 medium. Every week they were filtered to eliminate bigger clumps until quite homogeneous cells were obtained. For the following subculturing, two mL of packed cells were transferred into 50 mL fresh medium every 7 days (Cimini et al., 2018). Cells at 3 days after subculture were treated by adding 50, 100, or 150 mM NaCl to culture medium and used for the following experiments. Three independent experiments have been performed for each treatment to determine the growth capabilities of the two cell lines under salt stress, cells were filtered and the fresh and the dry weight were measured.

The osmotic stress contribution to cell death has been assessed by comparing the treatments with 100 mM NaCl and 200 mM D-mannitol. Cell death have been measured after 4 days of treatment. Five replicates have been performed.

For oxidative stress treatment, 14 mM glucose plus 1 U ml^-1^ GOX were added to 3 day-old cell suspensions in three independent experiments.

For glutathione pre-treatment, 3 mM GSH was added to 2 day-old cell suspensions, 1 day before 100 mM NaCl treatment. Three independent experiments were performed.

### Cell Viability

Cell death was evaluated by a spectrophotometric assay of Evans blue stain retained by cells according to Gaff and Okong’o-Ogola (1971) with minor modifications. Briefly, in three independent experiments for each treatment, one mL of cell suspension was sampled from the cultures at desired intervals. Evans blue dye solution was added to the cells to a final concentration of 0.05% and incubated for 15 min at room temperature, followed by washes with water until the washing showed no significant blue color. The washed cells were then added to 50% methanol containing 1% (w/v) sodium dodecyl sulfate and incubated at 55°C for 30 min. Absorbance was measured spectrophotometrically at 600 nm. The resulting OD was compared to the absorbance of cells boiled for 45 min, which are considered as 100% dead.

### DNA Laddering

Briefly, 1 g of cells was ground to a fine powder in liquid nitrogen. Hexadecyltrimethyl ammonium bromide (CTAB) isolation buffer (2% w/v CTAB, 1.4 M NaCl, 20 mM EDTA, 100 mM Tris–HCl, pH 8.0) at 60°C, freshly supplemented with 10 mM mercaptoethanol, was added to broken cells and mixed thoroughly. The mixture was incubated at 60°C for 40 min. Nucleic acids were extracted with equal volume of phenol and chloroform–isoamyl alcohol (24:1 v/v) and centrifuged at 3000 *g* for 15 min. DNA was incubated with 0.7 volumes of ice-cold isopropanol for 30 min at -20°C and centrifuged at 4000 *g* for 15 min at 4°C. The nucleic acid pellet was washed with an ice-cold 70% v/v ethanol and centrifuged for 5 min. The pellet was air dried and dissolved in 300 μL TE buffer (10 mM Tris–HCl, pH 8.0, 1 mM EDTA). DNase-free RNase A (0.1 μg μL^-1^) was added and the mixture incubated at 37°C for 30 min. DNA was separated on 2% agarose gel stained with ethidium bromide with equal amounts of 30 μg DNA per lane. Three independent experiments were performed for each treatment and the results of one of them is shown as representative in this paper.

### Hydrogen Peroxide Determination

Extracellular H_2_O_2_ was measured in culture medium as described by Bellincampi et al. (2000), with minor modifications in three independent experiments for each treatment. Briefly, 500 μL of the medium was added to an equal volume of assay reagent (500 μM ferrous ammonium sulfate, 50 mM H_2_SO_4_, 200 μM xylenol orange, and 200 mM sorbitol) and incubated for 45 min in the dark. The H_2_O_2_-mediated oxidation of Fe^2+^ to Fe^3+^ was determined by measuring the A560 of the Fe^3+^-xylenol orange complex. A calibration curve obtained by measuring the A560 of H_2_O_2_ standards allowed the conversion of the absorbance values into concentration estimates. All reactions were carried out at least in duplicate, and their reproducibility was checked. Values are expressed as μmoles of H_2_O_2_ per gram of cells Dry Weight (DW).

Intracellular H_2_O_2_ production was measured using dihydrorhodamine123 (DHR123; Sigma-Aldrich, Germany) as a probe in three independent experiments for each treatment. One ml of cell suspension was incubated with 20 μM DHR123 for 15 min in a rotating shaker, and then cells were washed three times with 1 mL of N6 medium. For fluorescence analyses, cells were imaged under a fluorescence microscope (DM5000, Leica Microsystems, Germany) with a I3 filter. About 100 images containing at least 10 cells were analyzed for each timepoint and treatment using ImageJ2 (Rueden et al., 2017). The total fluorescence was measured using the “Analyze particles” plug-in and averaged over the total area. In order to estimate the endogenous level of H_2_O_2_ of B and VN lines, cell extracts were obtained. At this purpose, B and VN cultured cells were pre-incubated with 20 μM DHR123 for 15 min, washed, harvested and homogenized in 50 mM Tris-HCl pH 7.5 buffer. H_2_O_2_ was quantified by using a spectrofluorometric standard curve (λ excitation = 490 nm; λ emission = 524 nm) obtained adding known amounts of H_2_O_2_ to the cell extracts.

### Determination of Antioxidant Activity and ASC and GSH Levels

The antioxidant activity was measured in B and VN cells under control and treatment conditions as reported in Locato et al. (2008). In particular, the antioxidant activity was measured by the 2,2′-azino-bis-3-ethylbenzthiazoline-6-sulphonic acid (ABTS)/horseradish peroxidase decolouration method in three independent experiments. This method enables to measure hydrophilic antioxidant and lipophilic antioxidant activity. Briefly, cells (0.3 g) were ground in a mortar in liquid nitrogen with 1.2 mL of 50 mM sodium phosphate buffer (pH 7.5) for the determination of the hydrophilic antioxidant activity and with 1.2 ml of ethanol for that of lipophilic one. The homogenates were centrifuged at 4000 *g* for 10 min at 4°C and the capability of the supernatants to scavenge the ABTS radical cations was compared with a standard dose–response curve obtained using 6-hydroxy-2,5,7,8-tetramethylchroman-2-carboxylic acid (Trolox) and was expressed as micromoles of Trolox equivalent per gram fresh weight. Being a hydrophobic compound, to dissolve Trolox in hydrophilic environment the standard solutions were sonicated for 30 min.

In three independent experiments for each treatment, cells (0.5 g) were collected by filtration and homogenized by adding four volumes of cold 5% (w/v) meta-phosphoric acid at 4°C for ASC and GSH assay. The homogenates were centrifuged at 14000 *g* for 15 min at 4°C and the supernatants were collected and used for the analysis of ASC and GSH according to Locato et al. (2015). In particular, the glutathione pool was assayed utilizing aliquots of 0.2 ml of supernatant neutralized with 0.3 ml of 0.5 M phosphate buffer (pH 7.5). For glutathione disulfide (GSSG) assay, the GSH was masked by adding 20 μl of 2-vinylpyridine to the neutralized supernatant, whereas 20 μl of H_2_0 was added in the aliquots utilized for total glutathione pool (GSH plus GSSG) assay. Glutathione content was measured in 1 ml of reaction mixture containing 0.2 mM NADPH, 100 mM phosphate buffer (pH 7.5), 5 mM EDTA, 0.6 mM 5,5′- dithiobis (2-nitrobenzoic acid), and 0.1 ml of sample obtained as described above. The reaction was started by adding 3 units of glutathione reductase and was monitored by measuring the change in absorbance at 412 nm during 1 min. GSH was estimated as the difference between the amount of total glutathione and that of GSSG. A standard curve for GSH in the range of 0–70 μM was prepared.

### Enzyme Assays

In three independent experiments for each treatment, cells (0.5 g) were ground in liquid nitrogen and homogenized at 4°C in an extraction buffer (50 mM Tris-HCl pH 7.5, 0.05% cysteine, 0.1% bovine serum albumin). Homogenates were centrifuged at 14000 *g* for 15 min at 4°C and the supernatants were used for the analyses of enzyme activity. Protein contents were determined according to Bradford (1976) using bovine serum albumin (BSA) as a standard.

The activity of SOD (superoxide:superoxide oxidoreductase, EC 1.15.1.1) was measured according to Beauchamp and Fridovich (1971) using the reduction of nitro blue tetrazolium as detector of O^2-^ produced via the riboflavin photoreduction. One SOD unit was defined as the amount needed to cause half-maximal inhibition of this reaction. Measurements were carried out at 560 nm.

The activity of ascorbate peroxidase (APX; L-ascorbate: hydrogen peroxide oxidoreductase, EC 1.11.1.11), glutathione reductase (GR; NADPH: glutathione disulfide oxidoreductase, EC 1.6.4.2) monodehydroascorbate reductase (MDHAR; NADH: ASC free radical oxidoreductase, EC 1.6.5.4) and dehydroascorbate reductase (DHAR; GSH: dehydroascorbate oxidoreductase, EC 1.8.5.1) were measured according to de Pinto et al. (2000). Catalase (CAT; hydrogen-peroxide: hydrogen-peroxide oxidoreductase, EC 1.11.1.6) activity was measured according to Veljovic-Jovanovic et al. (2001) and Sgobba et al. (2015).

### Analysis of Gene Expression by qPCR

In three independent experiments for each treatment, 1 g of cells was ground to a fine powder in liquid nitrogen and 1 ml TRIZOL was added. After centrifugation at 5 min 14000 *g*, the supernatant was added with 200 μl chloroform and centrifuged again. The upper phase with the RNA was transferred to a new tube and chloroform extraction was repeated. The resulting upper phase was incubated with 0.7 volumes of cold isopropanol at -20°C for 20 min and then centrifuged for 10 min at 14000 *g*. The pellet was washed twice with 500 and 150 μl of 70% ethanol, air-dried and resuspended in 30 μl of water. RNA concentrations were measured using a Nanodrop ND-1000 spectrophotometer (Nanodrop Technologies, Rockland, DE, United States) and 5 μg of RNA was treated with RQ1 DNase (Promega) according to the manufacturer’s protocols. RNA was finally precipitated with 2.2 μl of CH_3_COONa 3M pH 5.2 and 60.5 μl of ethanol and incubated for 30 min at -20°C. After centrifugation the pellet was washed with 70% ethanol, air-dried, resuspended in 5 μl of water and used for first-strand cDNA synthesis using the SuperScript II Reverse Transcriptase (Thermo Fisher) according to user instructions.

qPCR for the K^+^ and Na^+^ channels and transporters encoding genes analyzed in this study and for the SODCC2, and CAT A genes was performed using the QuantStudio 12K Flex real-time PCR system and OpenArray technology (Thermo Fisher Scientific, CA, United States), following the manufacturer’s instructions. TaqMan^®^ OpenArray^®^ Real-Time PCR Plate with Custom Gene Expression Assays (Supplementary Table S1) was designed and purchased from Thermo Fisher Scientific.

qPCRs for α-DOX2, SERF1, APX1, APX2 and CAT B genes were performed in a 7500 Real-time PCR System (Life Technologies) using the SYBR Green technology of GoTaq qPCR Master Mix (Promega) using specific primers (Supplementary Table S2).

The results were analyzed using the ΔΔ*C*_T_ method (Livak and Schmittgen, 2001).

### Ionic Analysis

Samples were digested with concentrated HNO_3_ in a microwave system. The elements concentration was determined by inductively coupled plasma ICP-OES, Ciros Vision EOP (Spectro A. I. GmbH, Kleve, Germany) in three independent experiments for each treatment. Elements quantifications were carried out using certified multi-element standards.

### Data Analysis

Data are presented as mean ± standard error (SE) from three independent experiments. The statistical significance was tested using Wilcoxon–Mann–Whitney’s test (*p* < 0.01) or one-way analysis of variance (ANOVA; *p* > 0.05) combined with Tukey’s honestly significant difference (HSD) *post hoc* test. The statistical analyses were performed using the GraphPad Prism v4.02 software (ANOVA) and STATISTICA (TIBCO Software Inc.).

## Results

### Characterization of Suspension Cell Cultures Produced From Two Rice Varieties That Differ in Terms of Salt Sensitivity

In order to study the mechanisms of salt tolerance at the cellular level, suspension cell lines were established from each of two Italian rice varieties showing contrasting salt sensitivity (Formentin et al., 2018), Baldo (B) as tolerant and Vialone Nano (VN) as sensitive. Both are intensively cultivated varieties of great economic importance for local (VN) and export (B) markets.

Two different cell lines obtained from each cultivar were tested for salt sensitivity and all of them showed the same pattern of sensitivity/tolerance observed in plants. In this report, the results obtained on two representative cell lines (indicated as B and VN) are shown. When results were not consistent among specific lines derived from the same variety, differences were reported.

**FIGURE 1:**
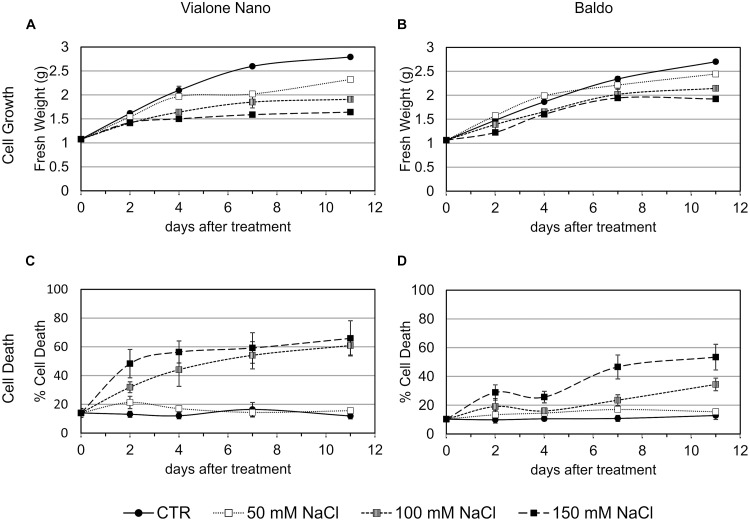
Cell growth and cell death of B and VN suspension cultured cells in presence and absence of NaCl. Three-day-old VN **(A,C)** and B **(B,D)** cells were treated with different concentrations of NaCl. Closed circles – straight line, Control; open squares – dotted line, 50 mM NaCl; gray squares – small-dashed line, 100 mM NaCl; closed squares – dashed line, 150 mM NaCl. **(A,B)** Fresh weight (g) of cells at different times after treatment. **(C,D)** Cell death percentage (Evans blue staining). Values represent the mean ± SE of three independent experiments performed in triplicate.

The two rice cell lines (B and VN) were exposed to several salt concentrations (50, 100, and 150 mM NaCl) and growth parameters along with measurements of cell death were evaluated (Figure 1). By comparing the growth curves of the two cell lines, differences in salt sensitivity were observed (Figures 1A,B). Measuring the percentage of cell death at different times during subculture cycles showed that VN cells reached 50% after 2 and 4 days, depending on the salt concentration (150 and 100 mM NaCl, respectively; Figure 1C). B cells, on the other hand, showed a smaller increase in the amount of cell death at 2 days. Cell viability remained almost stable up to 4 days of treatment, after which cell death increased again, reaching 30 and 50% at 10 days in the presence of 100 and 150 mM NaCl, respectively (Figure 1D). In both cell lines, no significant differences in cell viability were observed in the presence of 50 mM NaCl. Being the salt stress made of two components, osmotic and ionic, we tested whether the observed cell death was salt specific. The addition of D-mannitol in a concentration causing the same osmotic stress as 100 mM NaCl, induced a little increase in cell death respect to the control cells at 4 days of treatment, thus suggesting that the ion toxicity is contributing greater than osmotic stress to cell death (Supplementary Figure S1).

**FIGURE 2:**
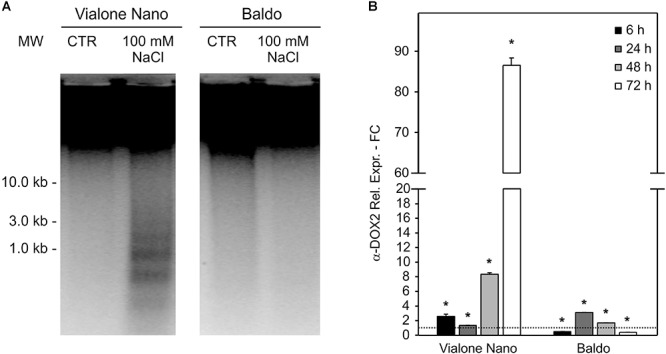
Programmed cell death features in salt sensitive VN and B resistant cells. Oligonucleosomal DNA fragmentation **(A)** and high expression of a-DOX2 **(B)** are present only in dying cells. Values represent the mean ± SE of three independent experiments. Asterisks indicate significative differences with respect to control samples (*p* < 0.01, Wilcoxon–Mann–Whitney’s test, Marx et al., 2016).

To better define the nature of the cell death induced by salt stress, DNA laddering, a well-known hallmark of PCD, was evaluated. DNA oligonucleosomal fragmentation was detected in VN cells after 7 days of treatment in the presence of 100 mM NaCl, when the cell death percentage reached almost 60%. In contrast, genomic DNA laddering in B cells remained undetectable, as only 20% of cell death occurred at that time point (Figure 2A).

Along with DNA laddering, the expression profile of *fatty acid alpha-oxidase* (or α-dioxygenase 2 *α-DOX2*), known for being associated with PCD (Koeduka et al., 2005), was analyzed. In VN cells, the expression level of α*-DOX2* highly increased, showing an eightfold increase at 48 h, and an almost 90-fold increase at 72 h. On the other hand, in the B cell line, a slight transient peak followed by a down regulation was observed at 24 h (Figure 2B).

**FIGURE 3:**
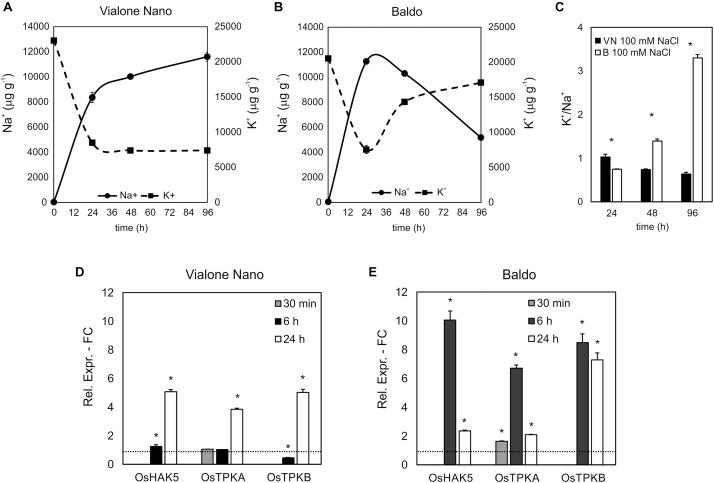
[K^+^] and [Na^+^] content and ion channel genes regulation. K^+^ **(A)** and Na^+^ **(B)** content in treated (100 mM NaCl) and untreated cells was measured, and the [K^+^]/[Na^+^] ratio was calculated **(C)**. **(D,E)** Expression profile of genes involved in K^+^ uptake in the cytosol. Values represent the mean ± SE of three independent experiments. Asterisks indicate significantly different levels (respect to the control in **D,E**, or between the two cell lines in **C**) according to Wilcoxon–Mann–Whitney’s test (*p* < 0.01).

Vialone Nano and B cell cultures were further characterized by evaluating their [K^+^]/[Na^+^] ratio, a trait considered to be predictive of salt tolerance/sensitivity (Munns and Tester, 2008). In both cell lines, the K^+^ content decreased after 24 h of salt treatment, and Na^+^ content increased strongly. The alteration in the levels of Na^+^ and K^+^ remained constant in VN. However, in the tolerant variety, intracellular K^+^ concentration increased again in the following hours, and Na^+^ levels progressively decreased (Figures 3A,B). Accordingly, in the tolerant variety, the [K^+^]/[Na^+^] ratio increased following the salt treatment, whereas in VN, the ratio decreased slightly (Figure 3C).

The maintenance of a high [K^+^]/[Na^+^] ratio is a low-cost strategy in salt response (Volkov, 2015). K^+^ can both be uptake from the medium, through potassium transporters or channels located at the plasma membrane, or released from the vacuole, where the ion is present at millimolar concentrations, through vacuolar outward channels (Volkov, 2015). We investigated the expression profiles of genes belonging to families of ion channels/transporters, focusing on those probably implicated in K^+^ uptake (OsHAK5; Horie et al., 2011) or release to the cytosol (*OsTPKa* and *OsTPKb*; Isayenkov et al., 2011). In agreement with the ionic data, all three genes were already highly upregulated after 6 h of salt treatment in the tolerant variety, whereas they appeared only slightly upregulated later on (24 h) in the sensitive variety (Figures 3D,E).

On the other hand, no differences between the two cell lines were observed in the expression profile of the vacuole-localized Na^+^/H^+^ antiporter (*OsNHX1*) (Barragán et al., 2012) and plasma membrane Na^+^/H^+^ antiporter (*OsSOS1*) (Martínez-Atienza et al., 2007) under control conditions and after salt treatment (Supplementary Figure S2).

### H_2_O_2_ as a Signal Molecule Induced by Salt Stress

Salt stress is known to induce H_2_O_2_ production (Gill and Tuteja, 2010). Depending on the levels, H_2_O_2_ can be involved in signaling pathways. Under threshold levels, H_2_O_2_ acts like a signaling molecule by activating defense and survival mechanisms (Baxter et al., 2014; Farnese et al., 2016). However, when present at higher concentrations, the oxidative-damaging character of this ROS prevails, and cell death occurs (de Pinto et al., 2006; Locato et al., 2016). H_2_O_2_ content was thus investigated in the rice cell lines over time.

**FIGURE 4:**
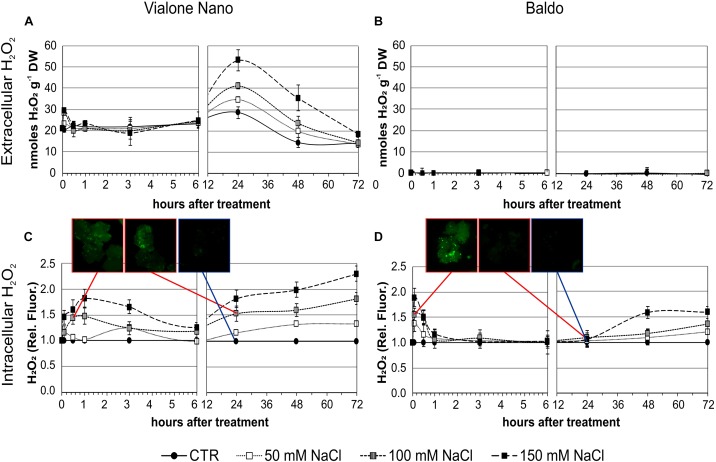
H_2_O_2_ produced by rice suspension cell cultures of VN and B and measured at different time points after salt treatment. Closed circles – straight line, Control; open squares – dotted line, 50 mM NaCl; gray squares – small-dashed line, 100 mM NaCl; closed squares – dashed line, 150 mM NaCl. **(A,B)** H_2_O_2_ released in the medium; DW, Dry weight. **(C,D)** Intracellular H_2_O_2_ measured using the DHR-123 method; values were normalized against the levels of control cells, which were given a value of 1 and therefore have no SD. Insets are representative images of stained cells at different timepoints. Values represent the mean ± SE of three independent experiments.

In VN, a basic level of H_2_O_2_ (approximately 20 nmoles g^-1^ DW) was measured in the medium of untreated cells, whereas in salt-treated cells, two distinct peaks of H_2_O_2_ were observed, with the first burst at 5 min (for 100 mM and 150 mM NaCl) followed by a sustained second dose-dependent peak at 24 h. Notably, H_2_O_2_ production occurred in a dose-dependent manner (Figure 4A).

In the two B cell lines, no increase in extracellular H_2_O_2_ was observed after salt treatment (Figure 4B) despite some differences were observed in the basal levels of the two (Supplementary Figure S3).

Using the dihydrorhodamine 123 (DHR123) fluorescent probe, the intracellular H_2_O_2_ level was investigated under the same experimental conditions. Interestingly, the level of intracellular H_2_O_2_ as revealed by dye intensity was significantly higher in VN cells than in B ones in control conditions (929 ± 124 and 687 ± 18 nmol/g FW, respectively). After 100 and 150 mM NaCl treatment, VN cells showed a broad peak of intracellular H_2_O_2_ starting at 1 h of treatment, and decreasing after 3–4 h. In the following hours, a progressive dose-dependent increase in H_2_O_2_ levels occurred in these cells (Figure 4C).

In the B more tolerant cell line, salt treatment induced a very narrow early peak of intracellular H_2_O_2_ production, which reached its maximum level at 5 min of treatment and was dose dependent. Later there was a slow increase, which was significantly higher than the control at 48 h with 150 mM NaCl and at 72 h with 50 and 100 mM NaCl (Figures 4C,D).

As the rice transcription factor gene *SERF1* is involved in the salt-specific and H_2_O_2_-dependent signaling (Schmidt et al., 2013), its expression profile was analyzed in order to understand whether the different “signature” of internal H_2_O_2_ observed in the two cell lines (Figures 4C,D) led to differences in the salt signaling pathway.

**FIGURE 5:**
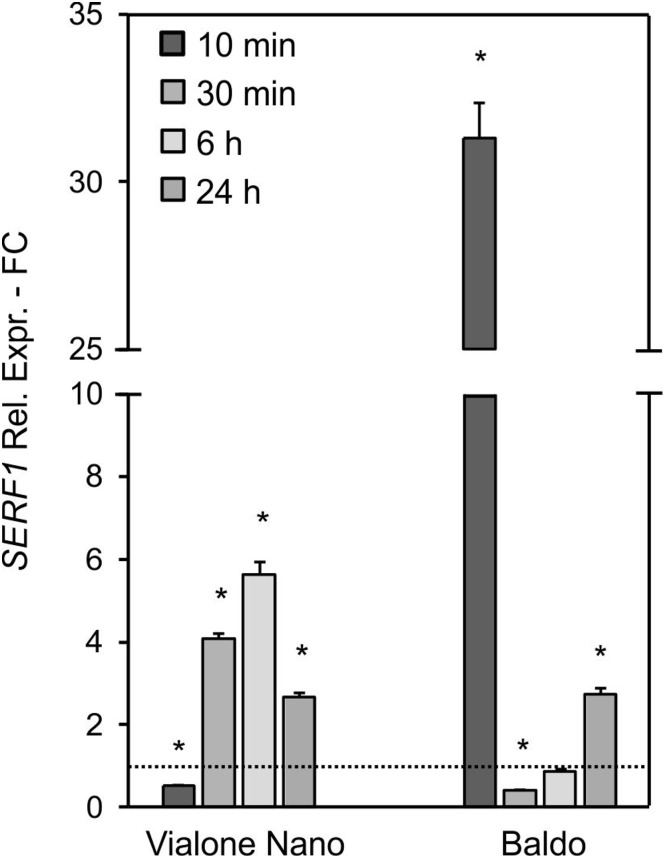
Relative expression (fold change) of the SERF1 gene at different time points after treatment with 100 mM NaCl in VN and B. Values represent the mean ± SE of three independent experiments in duplicate. The asterisk indicates values that are significantly different from those of untreated according to Wilcoxon–Mann–Whitney’s test (*p* < 0.01).

In B cells, a high transient increase in the *SERF1* transcript level (more than 30-fold) was observed within 10 min of salt stress (Figure 5), immediately after the first intracellular H_2_O_2_ peak (Figure 4D). Regarding the VN cells, a much lower expression peak was reached at 6 h after salt treatment (Figure 5), reflecting the shift in H_2_O_2_ production, as shown in Figure 4C.

These results suggested that the different intracellular H_2_O_2_ profiles observed in the two rice cell lines led to a different expression pattern of *SERF1*.

### ROS-Scavenging Systems

In order to use H_2_O_2_ as a signaling molecule, non-toxic levels must be maintained in a delicate balance between ROS production and ROS-scavenging pathways (De Gara et al., 2010; Suzuki et al., 2012; Foyer and Noctor, 2016).

Thus, ROS levels mainly depend on both the modulation in the activities of the ROS-scavenging enzymes and the regulation of their gene expression. Thus, the enzymatic and non-enzymatic systems involved in controlling H_2_O_2_ levels were analyzed.

**FIGURE 6:**
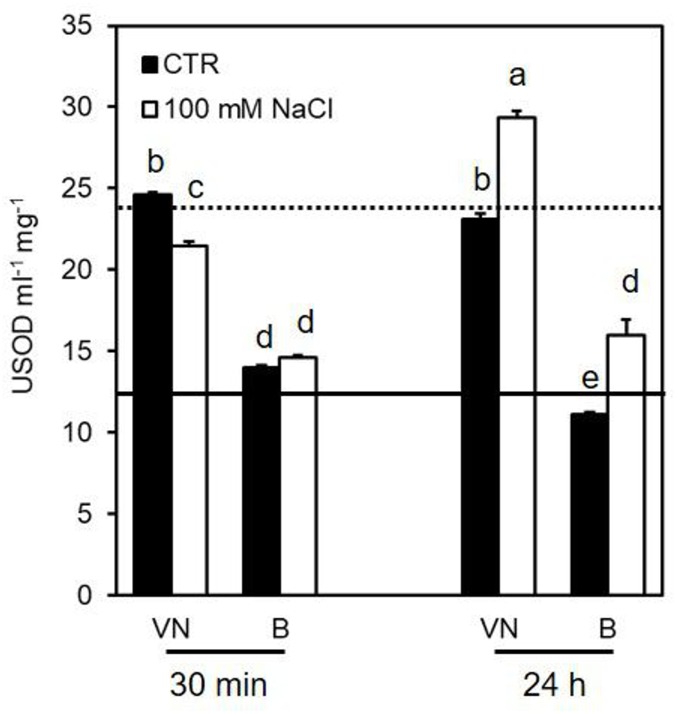
Effect of 100 mM NaCl on SOD activity determined at 30 min and 24 h after treatment. Values represent the mean ± SE of three independent experiments at least in duplicate. The lines represent average enzymatic activity in control conditions of VN (straight line) and B (dotted line). Different letters indicate significantly different activity levels according to one-way ANOVA (*p* < 0.05).

In untreated cells, VN showed a superoxide dismutase (SOD) activity higher than B (Figure 6). This is consistent with the higher amount of H_2_O_2_ observed in the VN cell cultures than in the B ones, in control conditions (Figure 4A). In the presence of NaCl, SOD activity showed a clear increase in both cell lines 24 h after treatment (Figure 6).

**FIGURE 7:**
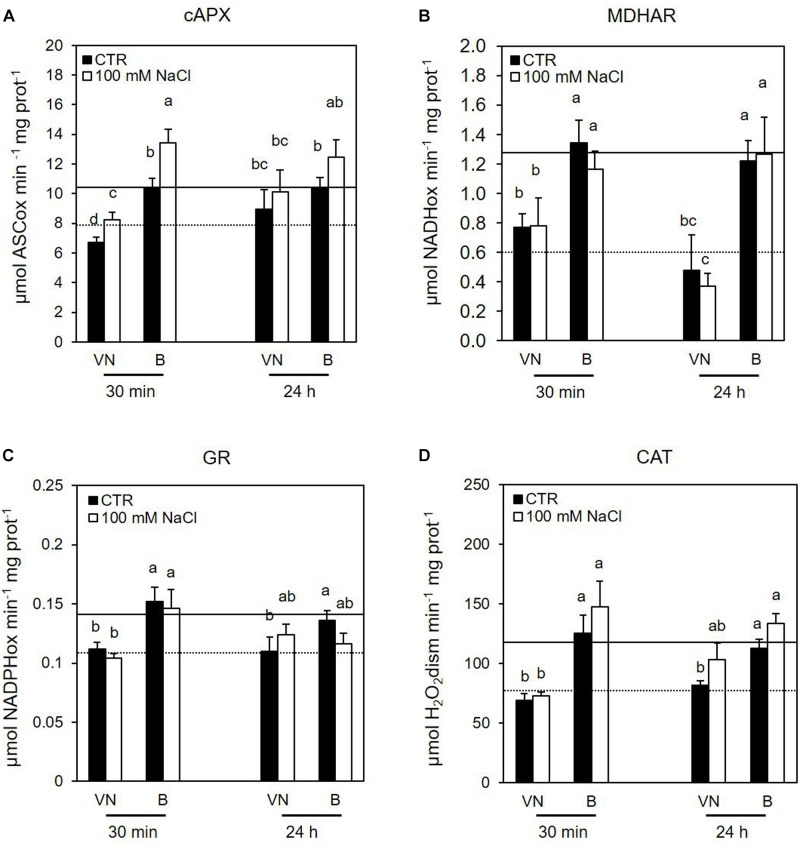
Effects of 100 mM NaCl on **(A)** cAPX, **(B)** MDHAR, **(C)** GR, and **(D)** CAT activity determined at 30 min and 24 h after treatment. Values represent the mean ± SE of three independent experiments. The lines represent average enzymatic activity in control conditions of VN (straight line) and B (dotted line). Different letters indicate significantly different activity levels according to one-way ANOVA (*p* < 0.05).

Several data support the key role of cytosolic ascorbate peroxidase (cAPX) in ROS homeostasis (Locato et al., 2009; de Pinto et al., 2013). Interestingly, the cAPX activity was higher in the more tolerant B cells, than in the more sensitive VN cells in control conditions over all treatment time (Figure 7A and Supplementary Figure S4). Figure 7 reports the results obtained at those treatment times (30 min and 24 h) when B and VN treated cells showed comparable viability; whereas the Supplementary Figure S4 reports the activity of redox enzymes assayed at 48 h from the treatment, when B and VN cell viability diverged. Salt stress induced a significant increase in this enzyme activity at 30 min, which was more evident for B than for VN (Figure 7A). A similar behavior was observed for catalase (CAT). In particular, CAT activity was always higher in B than VN in control conditions and showed a significant increment only in the salt–treated B cells at 48 h (Figure 7D and Supplementary Figure S4).

Considering the other enzymes involved in the ascorbate-glutathione (ASC-GSH) cycle, the tolerant variety showed a higher activity of monodehydroascorbate reductase (MDHAR) and glutathione reductase (GR) in control conditions, but no significant changes in the activities of these enzymes were observed after salt treatment (Figures 7B,C and Supplementary Figure S4). There were no significant differences in DHAR activity between the cultivars either in control conditions nor under salt stress (data not shown).

**FIGURE 8:**
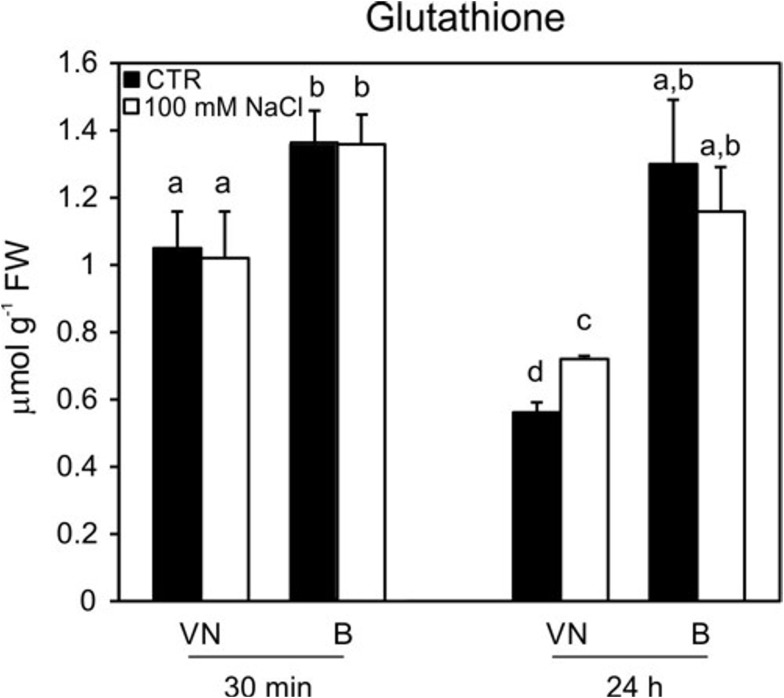
Glutathione content in treated (100 mM NaCl) and untreated cells. Changes in cellular levels of glutathione (reduced plus oxidized forms; GSH + GSSG) pools induced by 100 mM NaCl and determined at 30 min and 24 h after treatment. Values represent the mean ± SE of three independent experiments. Different letters indicate significantly different glutathione levels according to one-way ANOVA (*p* < 0.05).

The ASC and GSH contents were measured, as they represent the key metabolites involved in ROS scavenging (Foyer and Noctor, 2011). No significant differences in the content of ascorbate were observed in the two studied cell lines (Supplementary Figure S5). On the other hand, glutathione levels were lower in the VN cells than in the B cells at 30 min and 24 h in control conditions; in particular, at 24 h, the B cells had a content of glutathione that was almost double that of VN (Figure 8). However, at 48 h, the glutathione content of B cells became comparable with that observed in VN cells (0.61 ± 0.08 μmol/g FW and 0.64 ± 0.05 μmol/g FW, respectively). This trend probably depended on the general drop of antioxidant pools observed over growth curve of plant cultured cells (de Pinto et al., 1999; Pellny et al., 2009). In order to confirm the different endogenous antioxidant properties of the two cell lines, the assay of the antioxidant activity was performed. In control conditions, before the treatment, the hydrophilic antioxidant activity was higher in B cells than in VN ones (11.0 ± 0.54 vs. 8.1 ± 0.18 μmol Trolox equ/g FW) and remained constant until 48 h after treatment in B, whereas decreased of 29% in VN at the same time. No differences were observed in the lipophilic activity of the two lines in control conditions and after salt treatment (data not shown).

The expression of some genes coding for enzymes that regulate ROS levels was analyzed in both VN and B. We particularly focused on genes coding for those enzymes that had been previously shown to be involved in salt and oxidative stress responses in rice, such as SOD, cAPX and CAT.

**FIGURE 9:**
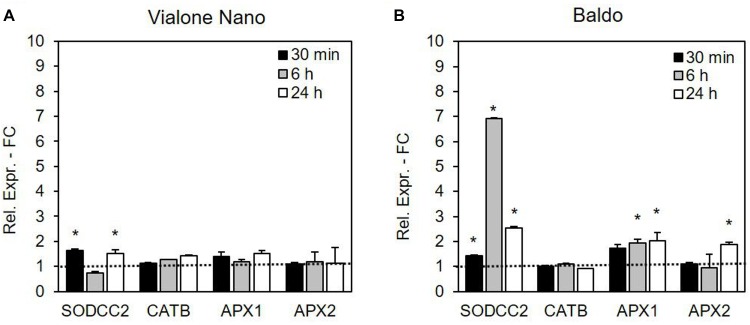
Relative expression (fold change) of the SODCC2, CATB, APX1, APX2, and GSTU6 genes at different times after treatment with 100 mM NaCl in **(A)** VN and **(B)** B. Values represent the mean ± SE of three independent experiments at least in duplicate. The asterisk indicates values that are significantly different from those of untreated cells according to Wilcoxon–Mann–Whitney’s test (*p* < 0.01).

Gene regulation was more rapid and efficient in the tolerant cell line B than in the sensitive one (Figure 9). In VN cells, the expression level of the SOD isoenzyme cytosolic copper/zinc-superoxide dismutase (*SODCC2*) showed an increase at 30 min and 24 h, and the increase in the gene expression was much more evident in the B cells, with a peak at 6 h. In relation to cAPX, *APX1* and *APX2* gene expressions were analyzed. In the more tolerant B cells, the expression levels of both genes increased over time, although more rapidly for *APX1* (already evident at 6 h) than for *APX2* (statistically higher than in the control cells only after 24 h of treatment). On the other hand, in the VN cells, no significant differences were detected in the expression levels of the two APX isoforms.

Among the CAT isoenzymes, the expression of the gene coding for *CATB* was not altered by salt stress in either of the two cell lines (Figure 9). No expression for *CATA* and *CAT1* was detectable in either cell line.

### Antioxidative Pathways and H_2_O_2_ Management

In order to verify whether the low level of antioxidative capability was a key point for VN sensitivity to salt stress, the glutathione levels were increased in these cells by adding 3 mM GSH to the culture medium 24 h before salt treatment.

The GSH pre-treated VN cells showed a higher content in glutathione (0.93 ± 0.2 μmol/g FW) respect to the not-enriched VN cells (0.67 ± 0.3 μmol/g FW) when exposed to 100 mM NaCl. The glutathione enrichment was accompanied by a progressive decrease in cell death, starting from day 4 of salt treatment (Supplementary Table S3).

**FIGURE 10:**
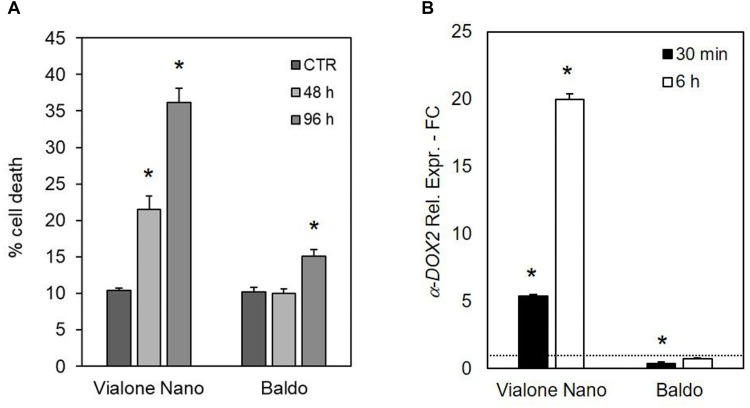
Effects of glucose/GOX on suspension cell cultures. **(A)** Percent survival of treated cells after 48 and 96 h of treatment with 14 mM glucose plus 1 U ml^-1^ GOX. **(B)** Expression profile of a-DOX2 gene after 30 min and 6 h of treatment with glucose/GOX. Values represent the mean ± SE of three independent experiments at least in duplicate. The asterisk indicates values that are significantly different from those of untreated cells according to Wilcoxon–Mann–Whitney’s test (*p* < 0.01).

The importance of different H_2_O_2_ management capabilities for salt sensitivity was confirmed by the different effects of exogenous H_2_O_2_ generation by the addition of glucose plus GOX (1 U ml^-1^) in the culture medium. As shown in Figure 10A, the VN cell death reached 22% after 48 h of treatment and almost 40% after 96 h. Notably, the salt tolerant variety showed a small increase in the percentage of cell death only 96 h after the addition of glucose/GOX to the culture medium. Increased levels of transcripts for a-DOX2 were also associated with VN undergoing cell death, but not with B cells, where a-DOX2 was also transiently downregulated at 30 min (Figure 10B).

**FIGURE 11:**
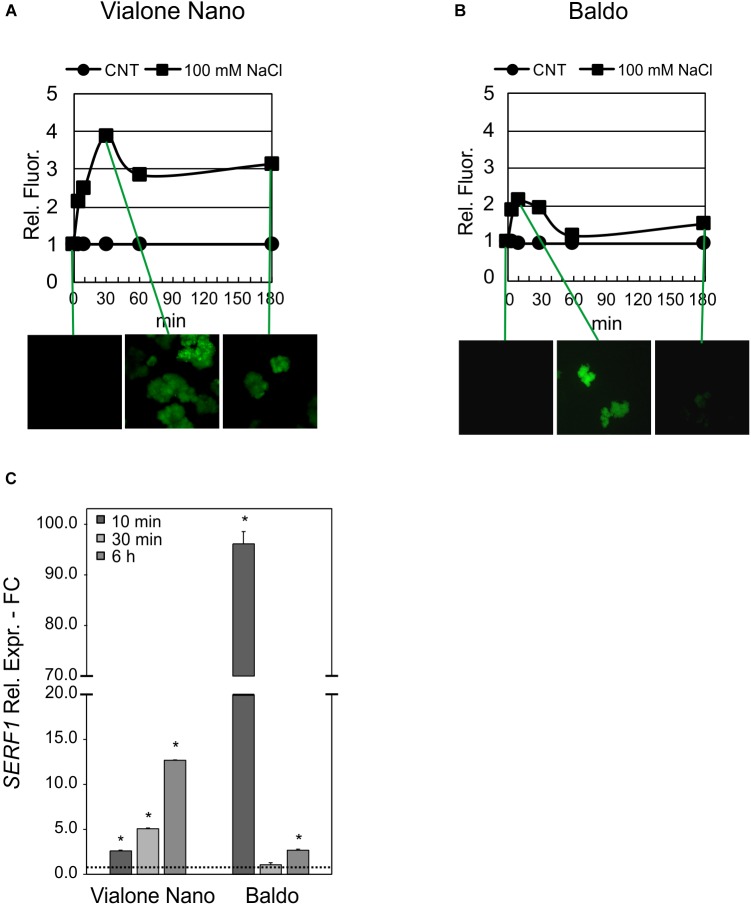
Intracellular H_2_O_2_ and signaling in glucose/GOX treated cells. The internal H_2_O_2_ production was imaged by using the DHR123 fluorescent dye **(A,B)** and the expression profile of *SERF1* **(C)**. Insets are representative images of stained cells at different timepoints. Values represent the mean ± SE of three independent experiments at least in duplicate. The asterisk indicates values that are significantly different from those of untreated cells according to Wilcoxon–Mann–Whitney’s test (*p* < 0.01).

The glucose/GOX treatment caused an increase in H_2_O_2_ in the media of both cultivars after 24 h. However, as shown in Table 1, B cells had a higher H_2_O_2_ – scavenging capability than the VN cells. A different profile in the intracellular H_2_O_2_ was also evident in the two cell lines. In VN cells, the H_2_O_2_ increase was higher than in B and more prolonged over time, and its decrease was much less pronounced (Figure 11A). Indeed, in B cells, a two-fold increase in the intracellular H_2_O_2_ occurred after 10 min of treatment. At 60 min, H_2_O_2_ was again found to have similar values to those of the control conditions, after which it increased again although without reaching the peak value (Figure 11B). The expression pattern of *SERF1* (Figure 11C) was also different in the two cell lines. Similarly to findings observed after salt stress, a strong and transient up-regulation occurred after 10 min of oxidative stress in B cells, while a much smaller and progressive increase occurred over time in VN cells. Notably, the oxidative stress induced by the glucose/GOX treatment had no effects on the expression profiles of ion transporter encoding genes (Supplementary Figure S6).

**Table 1:** Extracellular H_2_O_2_ content after treatment with glucose/GOX.

	nmoles H_2_O_2_ g^-1^ DW	
CTR	24.2 ± 3.1	N.D.
1UGOX	421.8 ± 6.8^∗^	44.1 ± 3.2^∗^
	Vialone Nano	Baldo

*H_2_O_2_ concentration was measured in the medium after 24 h in the absence (CTR) or presence of 14 mM glucose plus 1 U ml^-1^ GOX. Values represent the mean ± SE of three independent experiments. The asterisk indicates values that are significantly different from those of untreated cells according to Wilcoxon–Mann–Whitney’s test (p < 0.01)*.

## Discussion

Plants can differ greatly in their tolerance to salt stress, both among and within species. An initial characterization of Italian rice varieties identified VN and B as two cultivars with contrasting salt sensitivities: VN is more sensitive and B more tolerant (Bertazzini and Forlani, 2011; Formentin et al., 2018). To investigate salt-stress responses at the cellular level, suspension cell cultures from seeds of both varieties were generated. Notably, VN and B suspension cell cultures showed the same differences in salt sensitivity, as did whole plants.

We demonstrated that upon treatment with NaCl, B cells are able to set up specific tolerance mechanisms, whereas VN cells undergo cell death. Indeed, genomic DNA fragmentation (DNA laddering, Figure 2A), a hallmark of PCD (Hoeberichts and Woltering, 2003), and upregulation of α*-DOX2* (Figure 2B), a gene known for being associated with PCD (Koeduka et al., 2005) occur in salt-treated VN cells. Consistently with the different stress sensitivity, the expression of α*-DOX2* remained unaltered in B cells (Figure 2B), except for a slight and transient peak observed at 24 h, perhaps occurring as a common feature of stress response.

High salinity imposes two stress to cells: osmotic and ionic. The former takes place very early upon salt administration as a direct effect of the osmotic pressure increase at the plasma membrane. The latter takes hours or days depending on the speed of toxic ions accumulation and consequent metabolism impairment (Munns and Tester, 2008; Gupta and Huang, 2014). Though osmotic stress can contribute to the death of cells in the culture, we observed that the ionic component is more threatful and is accountable for the high death percentage measured after 4 days of treatment. In fact, the tolerance of B cells is related to the maintenance of the ion homeostasis, and in particular B cells, after an initial K^+^ loss, are able to increase [K^+^]/[Na^+^] ratio and restore survival conditions (Figure 3). This ability is a cost-effective trait of salt tolerance in plant cells (Senadheera et al., 2009). The analysis of the expression of genes involved in Na^+^ extrusion from the cytosol (*OsNHX1; OsSOS1*) did not reveal any difference between the two varieties; whereas, among the K^+^ transporters analyzed, the plasma membrane-located high-affinity K^+^ transporter (*OsHAK5*) (Horie et al., 2011) and the vacuolar-localized two-pore K^+^ channels (*OsTPKa* and *OsTPKb*) (Isayenkov et al., 2011), all implicated in increasing the [K^+^]_cyt_, exhibited different expression patterns between the two cell lines. These findings demonstrated that B cells actuated an ion homeostasis recovery obtained by controlling K^+^ concentration through the upregulation of *OsHAK5*, *OsTPK1a*, and *OsTPK1b* earlier and at more extent than VN cells (Figures 3D,E). Recently, the high capability to retain K^+^ in plant tissues has been proposed as a genetic trait conferring salinity tolerance (Chen et al., 2015; Wu et al., 2015). In particular, the increased K^+^ uptake in over-expressing *OsHAK1* rice plants seemed to sustain plant growth under K^+^ depletion, probably preserving the functionality of photosynthetic apparatus, normally reduced under NaCl stress (Chen et al., 2015).

These data indicate that salt stress induced different response pathways in the two cell lines, leading to a different cell fate: PCD or recovery. In order to better understand the signaling pathways leading to these different metabolic responses, the alterations relative to H_2_O_2_ and cellular redox homeostasis were studied in cell cultures of the two varieties exposed to salt stress.

In plants, ROS, which are produced by various metabolic pathways, are increased under salt stress (Apel and Hirt, 2004). Notably, in our study in B cells no increase in extracellular H_2_O_2_ was observed under salt treatment (Figure 4B), while there was a biphasic trend (5 min and 24 h peaks, Figure 4A), typical of PCD (De Gara et al., 2003), in the medium of VN sensitive cells. This difference was also reflected in the alterations of intracellular H_2_O_2_ levels occurring after salt treatment in the two varieties cell lines, with a clear difference in the shape and timing of the first early peak (Figures 4C,D) and in the extent of long-term H_2_O_2_ production. Indeed, VN cells responded to salt slowly by generating an initial large peak of H_2_O_2_ around 1 h, whereas B cells showed a quick response with a narrow H_2_O_2_ peak at 5 min. Over the following days, H_2_O_2_ continued to accumulate in a dose-dependent manner and to a much higher extent in sensitive compared to tolerant cells. These results seem to characterize the different response to salt stress of the two varieties, since salt stress induced an early peak of intracellular H_2_O_2_ also in roots of B plants with respect to VN roots (Formentin et al., 2018).

Although high levels of ROS induce cell death (de Pinto et al., 2006), a controlled and transient production of H_2_O_2_ has been associated with many signaling pathways, leading to stress tolerance (Jaspers and Kangasjarvi, 2010). Consistently, the decreased ROS production observed in *Arabidopsis* RBOHD1/2-RBOH1F/2 silenced plants lead to increased sensitivity to salt (Ma et al., 2012). Indeed, the expression of salt-responsive genes seems to be activated by ROS (Locato et al., 2018). Therefore, the transient increase in intracellular H_2_O_2_ observed in the tolerant variety highlights its signaling function in the activation of a salt defense response in B cells. The expression profile of *SERF1*, a gene involved in salt-induced and H_2_O_2_-mediated signaling processes (Schmidt et al., 2013), also followed the internal H_2_O_2_ profile, with a strong peak at 10 min in B cells and a large and late peak at 6 h in VN sensitive cells (Figure 5).

The different H_2_O_2_ levels and profiles detected in VN and B cells may depend on the different detoxification capacities of the two rice cell cultures. In order to assess this, gene expression and activities of enzymes involved in antioxidant systems were performed.

The expression analysis of genes encoding ROS-detoxifying enzymes showed that, under salt stress, B cells upregulated *SODCC2*, *APX1*, and *APX2* (Figure 9) more or earlier than VN cells. This suggests that the tolerant variety is more able to modulate the expression of genes aimed at controlling ROS levels than the sensitive variety. Accordingly, the ectopic expression of cytosolic APX and SOD has been found to increase the tolerance to salt stress of plum plants (Diaz-Vivancos et al., 2013). The salt-dependent upregulation of several ROS scavenging genes has also been reported in other rice varieties (Kim et al., 2007).

When these enzymes were analyzed in terms of their kinetic activities, no significant differences between the behaviors of the two varieties were induced by salt stress (Figures 6, 7). This apparent absence of the consistency between gene expression and enzyme activities can be explained by the two used experimental approaches: antioxidative activities were assessed as a whole, independently by the presence of different isoenzymes putatively differently regulated, whereas expression profiles were analyzed for genes encoding single isoenzymes.

Different timings in the processes occurring downstream of the gene expression, protein turnover and post-transcriptional modification, which affect enzymatic activity, could also be another reason for the lack of consistent results between these two experimental approaches. However, the presence of a higher redox control in B than in VN cells is also suggested by the different levels of enzyme activities involved in ROS scavenging which the tolerant variety already showed under control conditions. Notably, the activities of MDHAR, GR, CAT, and cAPX were already higher in the tolerant variety than in the sensitive variety under control conditions (Figure 7), as well as the glutathione content was significantly higher in B than in VN cells (Figure 8). When the activity of the mentioned redox enzymes were analyzed in B and VN seedlings, consistent results were obtained. In particular, cAPX and MDHAR activities were 30% higher in B than in VN, whereas CAT showed 15% higher activity in B than in VN seedlings (Ronci, Locato, De Gara personal communication). This further supports the innate higher capacity of ROS scavenging in B, which is important for inducing a higher tolerance to salt stress. It is also worth noting that when the glutathione level was enhanced in VN by its exogenous addition in the culture medium, salt-induced cell death was prevented, further confirming that innate levels of antioxidative defense are pivotal in tolerating this stress. Consistently, sulfur supplementation increased salt tolerance in mustard by reducing oxidative stress (Fatma et al., 2014, 2016).

The results of the exposure of the two cell cultures to oxidative stress imposed by the glucose/GOX-dependent H_2_O_2_ production in culture medium provide additional proof of the higher ROS-scavenging capacity of B cells. Indeed, the tolerant variety was able to scavenge the exogenous production of H_2_O_2_ more efficiently than the sensitive variety (Table 1), and to prevent cell death; whereas, the exogenous H_2_O_2_ production induced cell death in the sensitive VN line (Figure 10A). It is interesting to note that in spite of the constant and high production of ROS caused by the addition to culture medium of glucose/GOX, the intracellular H_2_O_2_ profiles looked different in the two rice lines. The tolerant cells were able to maintain the H_2_O_2_ signature observed under salt stress, with an early transient peak reaching its maximum at 10 min and disappearing in 1 h. On the other hand, the sensitive variety showed a peak with a maximum at 30 min, after which intracellular H_2_O_2_ persisted at a much higher level than in the control conditions. Again, the expression pattern of *SERF1*, the transcription factor induced by H_2_O_2_, was different in the two cell lines (Figure 11C), showing a similar trend to that observed in response to salt stress. Consistently, Hoang et al. (2015) reported that transgenic rice overexpressing anti-apoptotic genes showed reduced ROS accumulation and increased salt tolerance.

It is worth noting that in oxidative stress conditions but without salt stress, no induction in the expression of the three genes, *OsHAK5*, *OsTPK1a*, and *OsTPK1b*, contributing to the increase in [K^+^]_cyt_ were observed in the B tolerant cells. This result, which is in line with the absence of ionic stress, suggests that the signaling pathway involving the activation of *SERF1* is not the only one required for a correct metabolic adjustment which enables cells to counteract salt stress.

Overall these results show that in our experimental system, a difference in time, extent and intensity of the intracellular H_2_O_2_ production in response to salt stress, along with a different innate antioxidative activity/ROS scavenging capacity, could be responsible for the different fate and defense responses observed in the two cell lines (i.e., the induction of resistant mechanisms in B cells and the process of cell death in VN cells). At the same time, it is evident that B bases its resistance to salt stress on its better capacity to coordinate the co-presence of different stimuli (H_2_O_2_ and ionic stress in our case) and to trigger complex responses, which maintain redox and ionic homeostasis within cells.

## Author Contributions

EF, MZ, LDG, and FLS designed the research. EF, CS, MR, EB, PS, and BI performed the research. EF, CS, MR, and VL performed the data analysis, collection, or interpretation. EF, FLS, VL, and LDG wrote the manuscript.

## Conflict of Interest Statement

The authors declare that the research was conducted in the absence of any commercial or financial relationships that could be construed as a potential conflict of interest.

## Footnotes

1 http://www.fao.org/3/a-i4373e.pdf
