# Supplementary material for: H2O2 Signature and Innate Antioxidative Profile Make the Difference Between Sensitivity and Tolerance to Salt in Rice Cells
Source: Front Plant Sci. 2018 Oct 23;9:1549. doi: 10.3389/fpls.2018.01549 (PMC6206305; doi:10.3389/fpls.2018.01549)
Supplement: Supplementary file 1 [file Table_1.docx]

**Supplementary information**

**Table S1. List of Gene Expression Assays used in this work.**

| **Assay_ID** | **Gene_ID MSU** | **Gene_ID RAP** | **Gene_description** |
| --- | --- | --- | --- |
| AJ39RWU | LOC_Os07g01810 | Os07g0108800 | OsTPKb |
| AJ39RWY | LOC_Os01g70490 | Os01g0930400 | OsHAK5 |
| AJAAZRK | LOC_Os07g47100 | Os07g0666900 | OsNHX1 |
| AJQJB0J | LOC_Os03g54100 | Os03g0752300 | OsTPKa |
| AJS08CU | LOC_Os07g46990 | Os07g0665200 | superoxide dismutase [Cu-Zn] 2 (SODCC2) |
| AJY9Y72 | LOC_Os02g02400 | Os02g0115700 | catalase isozyme A |
| Os03619633_g1 | LOC_Os12g44360 | Os12g0641100 | OsSOS1 |
| Os03640561_s1 | LOC_Os03g08010 | Os03g0177400 | elongation factor 1-alpha (REFA1) |
| AIS09F9 | LOC_Os01g22490 | Os01g0328400 | ubiquitin-40S ribosomal protein S27a-1 (UBQ) |

**Table S2. List of primers for qPCR used in this work.**

| **Gene** | **Gene_ID** | **Primer_Forward** | **Primer_Reverse** |
| --- | --- | --- | --- |
| α-DOX2 | Os12g0448900 | 5'-AAATTAAAGGCCCCCTGCCA-3' | 5'-TGTGTCGTGCGATGAATCCT-3' |
| SERF1 | Os05g34730 | 5'-GAGTGAGGAGCTCATTGTTTACGA-3' | 5'-ACATCAAAATTTCCATGTCATCTA-3' |
| APX1 | Os03g0285700 | 5’-AAGACTACAAGGAGGCCCAC-3' | 5’-CAAGAGTACCACGGGCAATG-3' |
| APX2 | Os07g0694700 | 5’-CCTGATGCCACACAAGGTTC-3’ | 5’-TGTGACCACCAGAAAGAGCA-3’ |
| CATB | Os06g0727200 | 5’-AGAGAGCCTGCACATGTTCT-3' | 5’-AAACCCTCCATGTGCCTGTA-3' |
| UBQ | Os05g0160200 | 5'-TTCTACAAGGTGGACGACGC-3' | 5'-AGATCAGAGCAAAGCGAGCA-3' |

| **Experimental conditions** | **Cell death (%) over NaCl treatment time (days)** | | |
| --- | --- | --- | --- |
|  | II | IV | VII |
| Salt treated VN cells | 32 ± 3,0 | 43 ± 2,0 | 52 ± 4,0 |
| Salt treated VN cells enriched in GSH | 31 ± 2,8 | 35,8 ± 1,5 | 38,5 ± 3,1 |

**Table S3. Protective effect of GSH pre-treatment in VN cells challenged with 100 mM NaCl**

**
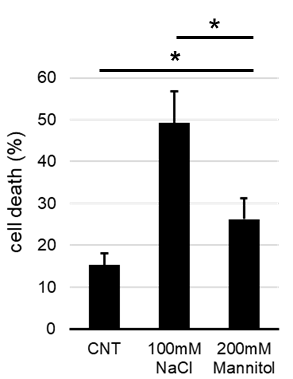
**

**Figure S1**. **Comparison between salt and osmotic stress.** Cell death of VN cells treated for 4 days. Values represent the mean ± SD of five replicates. Asterisks represent p<0.01.


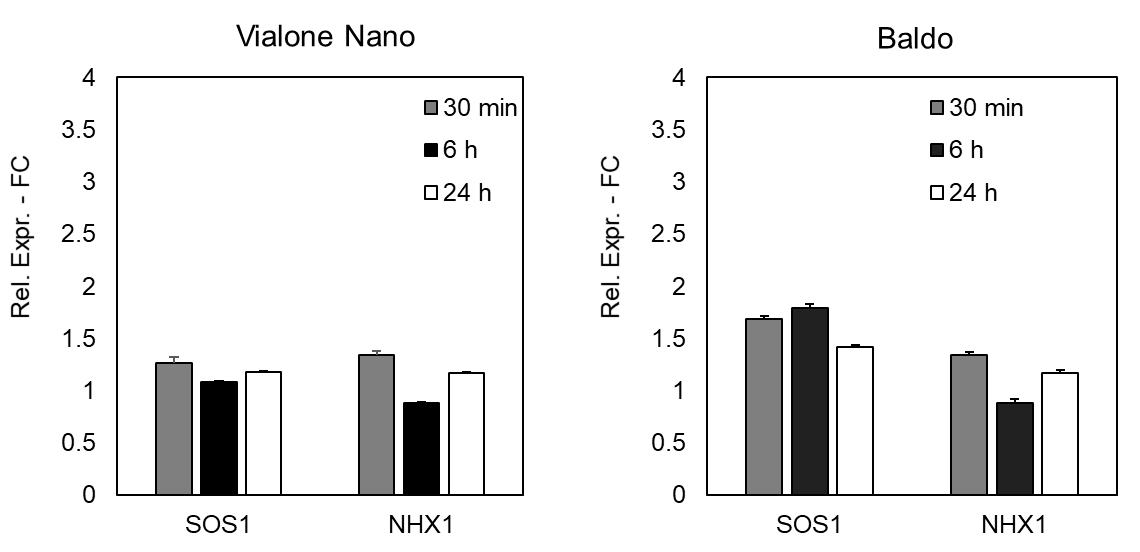


**Figure S2**. **Ion channel genes regulation.** Expression profile of genes involved in Na^+^ extrusion from the cytosol. Values represent the mean ± SE of three independent experiments (p>0.05).

**
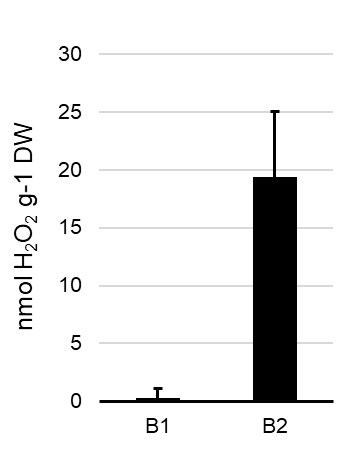
**

**Figure S3**. **Basal levels of H_2_O_2_ in the two Baldo cell lines used in this study.** External H_2_O_2_ was measured in control conditions at day 4 of subculture. Values represent the mean ± SD of five replicates.

**
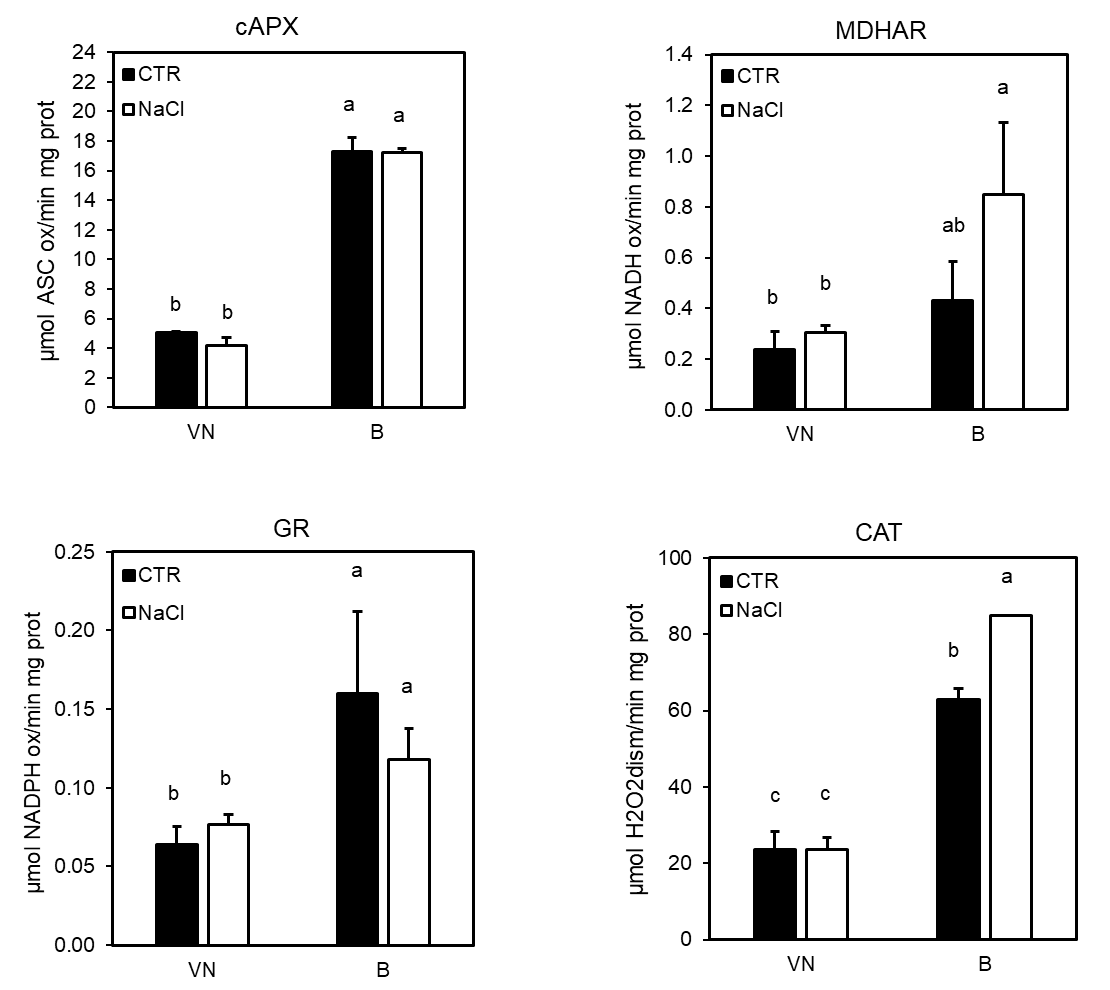
**

**D**

**C**

**B**

**A**

**Figure S4. Effects of 100 mM NaCl on (A) cAPX, (B) MDHAR, (C) GR and (D) CAT activity determined at 48 h after treatment.** Values represent the mean ± SE of three independent experiments. Different letters indicate significantly different activities according to one-way ANOVA (p<0.05).

**
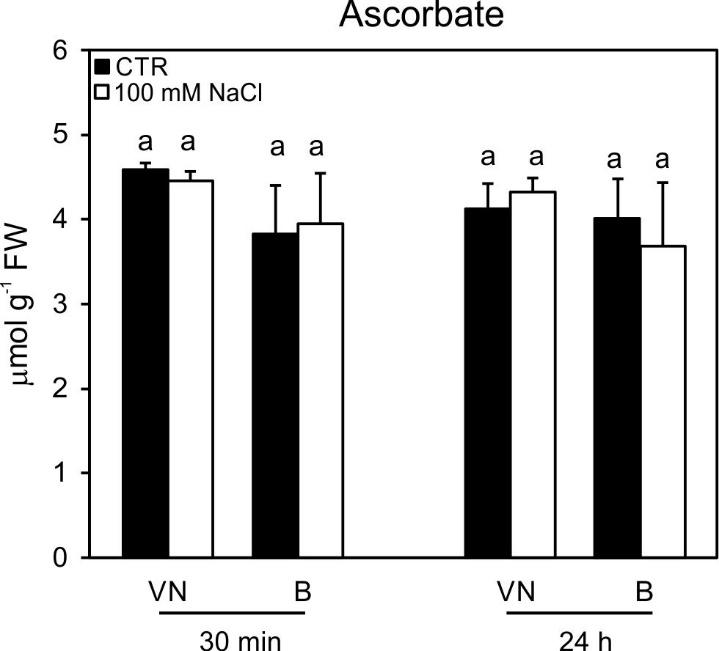
**

**Figure S5. Changes in cellular levels of ascorbate (ASC+DHA) pools** induced by 100 mM NaCl and determined at 30 min and 24 h after treatment. Values represent the mean ± SE of three independent experiments. Different letters indicate significantly different activities according to one-way ANOVA (p<0.05).

**
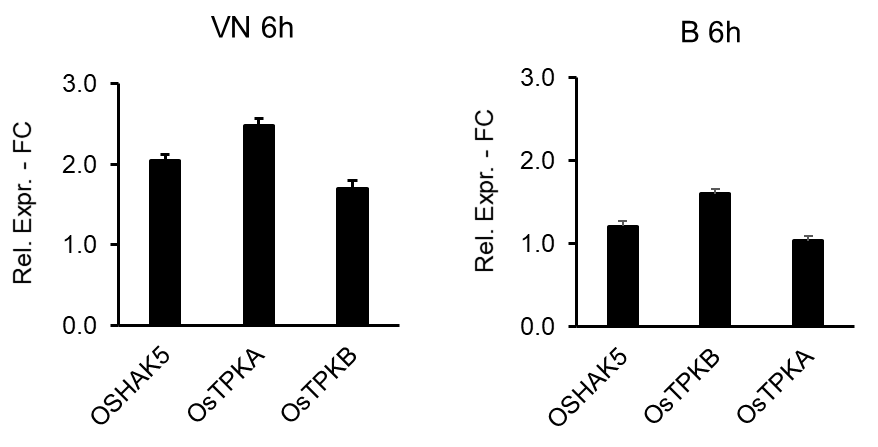
**

**Figure S6. Expression profile of genes involved in K^+^ transport after glucose/GOX treatment.** Values represent the mean ± SE of three independent experiments (p>0.05).
